# Extensive Analysis of *GmFTL* and *GmCOL* Expression in Northern Soybean Cultivars in Field Conditions

**DOI:** 10.1371/journal.pone.0136601

**Published:** 2015-09-15

**Authors:** Guangyu Guo, Kun Xu, Xiaomei Zhang, Jinlong Zhu, Mingyang Lu, Fulu Chen, Linpo Liu, Zhang-Ying Xi, Andreas Bachmair, Qingshan Chen, Yong-Fu Fu

**Affiliations:** 1 College of Agriculture, Northeast Agricultural University, Harbin, China; 2 MOA Key Lab of Soybean Biology (Beijing), National Key Facility of Crop Gene Resource and Genetic Improvement, Institute of Crop Sciences, Chinese Academy of Agricultural Sciences, 12 Zhongguancun Nandajie, Haidian District, Beijing, China; 3 Graduate School of Chinese Academy of Agricultural Sciences, Beijing, China; 4 College of Agronomy, Henan Agricultural University, Zhengzhou, China; 5 Dept. of Biochemistry and Cell Biology, Max F. Perutz Laboratories, University of Vienna, Vienna Biocenter, Dr. Bohr Gasse 9, A-1030 Vienna, Austria; University of Missouri, UNITED STATES

## Abstract

The *FLOWERING LOCUS T* (*FT*) gene is a highly conserved florigen gene among flowering plants. Soybean genome encodes six homologs of *FT*, which display flowering activity in *Arabidopsis thaliana*. However, their contributions to flowering time in different soybean cultivars, especially in field conditions, are unclear. We employed six soybean cultivars with different maturities to extensively investigate expression patterns of *GmFTL*s (*Glycine max FT*-like) and *GmCOL*s (*Glycine max CO*-like) in the field conditions. The results show that *GmFTL3* is an *FT* homolog with the highest transcript abundance in soybean, but other *GmFTL*s may also contribute to flower induction with different extents, because they have more or less similar expression patterns in developmental-, leaf-, and circadian-specific modes. And four *GmCOL* genes (*GmCOL1*/*2*/*5*/*13*) may confer to the expression of *GmFTL* genes. Artificial manipulation of *GmFTL* expression by transgenic strategy (overexpression and RNAi) results in a distinct change in soybean flowering time, indicating that *GmFTL*s not only impact on the control of flowering time, but have potential applications in the manipulation of photoperiodic adaptation in soybean. Additionally, transgenic plants show that *GmFTL*s play a role in formation of the first flowers and in vegetative growth.

## Introduction

Photoperiod sensitivity is an important trait, which allows crops to adapt to diverse latitudinal environments for flowering and maturation at a suitable season. In plants, many genes have been identified until now that are involved in the photoperiodic pathway of flowering. Two key genes, *CONSTANS* (*CO*) and *FLOWERING LOCUS T* (*FT*), form a central regulon (*CO-FT*) for flowering regulation [1]. The *CO-FT* regulon is a highly conserved module in many plants including *Arabidopsis* and crops, with diversification across a diverse groups of angiosperms [2, 3]. The regulon contributes to ecogenetic variation in a highly adaptive trait in different plants [4].

Even though *FT*, belonging to PEBP (**P**hosphatidyl **E**thanolamine-**B**inding **P**rotein) gene family [5, 6], is a highly conserved florigen across plants, *FT* and its like-genes in different versions may perform distinct functions in different plants [7]. For example, *Arabidopsis* has a main gene, *FT*, which controls photoperiodic flowering [8], while it homeolog *TSF* participates in flowering regulation in a special pathway that mediates cytokinin input [9]. *AtFT* and *AtTSF* are also involved in regulation of lateral shoot outgrowth [10]. In rice, two *FT* homologs, *Hd3a* and *RFT1*, control flowering in short day and long day conditions, respectively [11–13]. Maize has an *FT*-like family, including *ZCN8*, which is a good candidate of florigen [14]. The potato floral and tuberization transitions are controlled by two different *FT*-like paralogs (*StSP3D* and *StSP6A*) that respond to independent environmental cues [15]. Two *FT*s in *Populus* contribute flower initiation, but *FT1* functions in winter, while *FT2* in summer [16]. In tomato, *FT* ortholog *SFT* not only accelerates flowering in a dose-dependent manner, but also enhances fruit set [17, 18]. The *BvFT1* and *BvFT2* paralogs in beet act antagonistically on flowering regulation and growth habit [19]. *FT* also regulates *Arabidopsis* stomatal opening in a cell-autonomous mode [20], flower development [21] and inflorescence meristem stabilization [22]. The soybean genome contains at least six *FT* homeologs (*GmFTL1-6*), which show flowering activity in *Arabidopsis* [23–25]. Two of them, *GmFTL3* (a.k.a *GmFT2a*) and *4* (a.k.a. *GmFT5a*) (S1 Table) were experimentally confirmed to enhance flowering in soybean [26–28].

CO is a zinc finger transcriptional factor of the B-box family (BBX) family [29] and also conserved in the plant kingdom including algae [30–32]. During the evolutional history, *CO* family genes diverged with diverse functions but kept the photoperiodic-/circadian-regulated characters: *Chlamydomonas* growth [31], *Arabidopsis* [29] and rice [33] flowering, potato tuberization [34], the short-day-induced growth cessation and bud set occurring in the fall in aspen [4]. However, it is proven that not all *CO*-like genes regulate *FT* expression, and divergence of functions is widely spread across plants [3].

Soybean, originating from South China (lower latitude region), is a typical short-day plant for flowering induction. After genetic divergence, soybean underwent domestication (artificial selection) 6.000–9.000 year ago resulting in many cultivars, which adapted different environmental conditions in different areas [35]. In the soybean domestication, maturity is one of the important agronomic traits and closely related to flowering time. During the long history of soybean domestication, the sensitivity of soybean to the length of light irradiation was weakened. Thus, many soybean cultivars can flower in higher-latitude (northern, with long duration of sunshine) regions [36, 37]. For example, Kennong 18, a cultivar from Harbin (in northern of China, located at EL125°42′-130°10′ and NL44°04′-46°40′) can flower even under long-day photoperiod (18 hrs/6 hrs, light/dark) [38], indicating that northern soybean cultivars are much less sensitive to photoperiods and their critical-day-lengths are longer than that of their southern ancestors. The locus affecting the time of maturity in soybean was firstly designated as *E* locus by Owen [39]. Until now, there are at least ten loci (nine *E* and one *J* loci), which are reported to function in flowering and maturation in soybean: *E1* and *E2* [40], *E3* [41], *E4* [42], *E5* [43], *E6* [44], *E7* [45], *E8* [46], *E9* [47] and *J* [48]. *E1* encodes a protein containing a putative bipartite nuclear localization signal and a region distantly related to B3 domain [49], *E2* is a *Gigantea* (*GI*) gene [50], *E3* [51] and *E4* [52] are phytochrome A genes. These loci regulate flowering time through *GmFTL*s.

In this study, the detailed expression spectra of *FT*-like and *CO*-like genes under field conditions were analyzed to evaluate the expression divergences of different gene copies among different cultivars. In addition, both ectopic overexpression and silencing of *GmFTL* homologs in soybean were also employed to confirm *GmFTL* functions in photoperiodic flowering. The results show that all *GmFTL*s contribute to flowering control and *GmCOL*s regulate *GmFTL*s’ expression. Additionally, *GmFTL*s exhibit additional functions beyond flowering regulation.

## Results

### Northern soybean cultivars have much longer critical-day-length

**Fig 1:**
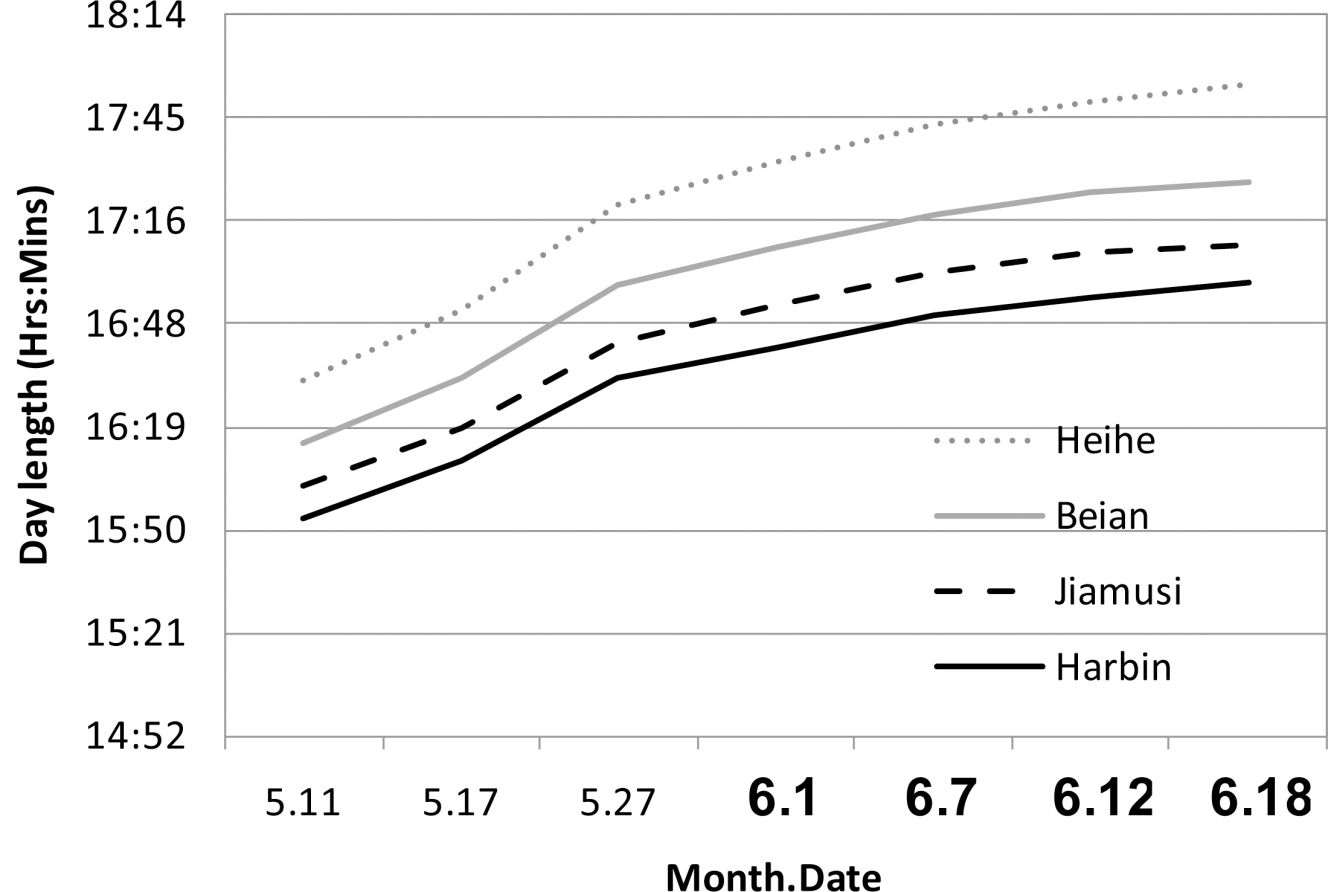
The day length at origin locations of different soybean cultivars during growth season. Day length data are from the Era Shuttle Calendar (http://if.ustc.edu.cn/~ygwu/calendar.html). All cultivars were planted on May 11 for sampling at different dates (bold letters on X-axis) at Harbin district, Heilongjian province, China.

To understand the relationship between soybean florigen gene *GmFTL*s and flowering habits in field conditions, we employed three northern groups (two cultivars for each) with different maturities (early, middle, and late) in local fields (Table 1). All cultivars show semi-determinate growth habits except Beidou 5, which is indeterminate. During the growth season, the local durations of sunshine ranges from 16 to 18 hours (Fig 1), which are typical long-day conditions for most of plants including soybean. It is well known that *Glycine max* cv Biloxi, a typical soybean cultivar for photoperiod study in the last century, has a critical day length of 13.5 hours [53]. Therefore, these data confirmed that the cultivars employed here were all photoperiod-hyposensitive. To compare their flowering response to day length, six oybean cultivars were grown in a Harbin region, where the day length was about 16.5 hours during the growth season and shorter than that of their original locations (Fig 1), to make sure all cultivars can flower and mature normally.The results showed that early cultivars had three fully-opened trifoliolates when flowering, whereas middle and late ones had four trifoliolates. Then, to investigate potential differences of different leaves in flowering induction, we harvested individual leaves according to developmental stages for real time quantitative PCR (RT-qPCR) analysis of genes, including *GmFTL*s, *GmCOL*s, and *E* genes.

**Table 1:** The information of cultivars used in this study.

Cultivars	Local region	Local longtitude/latitude	Maturity Group*	Maturity day*	Growth habit
Heihe 35	Heihe	EL127°53′/ NL50°22′	Early	91	Semi-determinate
Heihe 45	Heihe	EL127°53′/ NL50°22′	Middle	106	Semi-determinate
Beidou 51	Beian	EL126°50′/NL48°22	Early	94	Semi-determinate
Beidou 5	Beian	EL126°50′/NL48°22	Middle	115	Indeterminate
Hefeng 43	Jiamusi	EL130°35′/ NL46°83′	Late	123	Semi-determinate
Kenfeng 16	Jiamusi	EL130°35′/ NL46°83′	Late	120	Semi-determinate

*indicates the maturity in original location.

### Expression of *E* genes in different soybean cultivars

**Fig 2:**
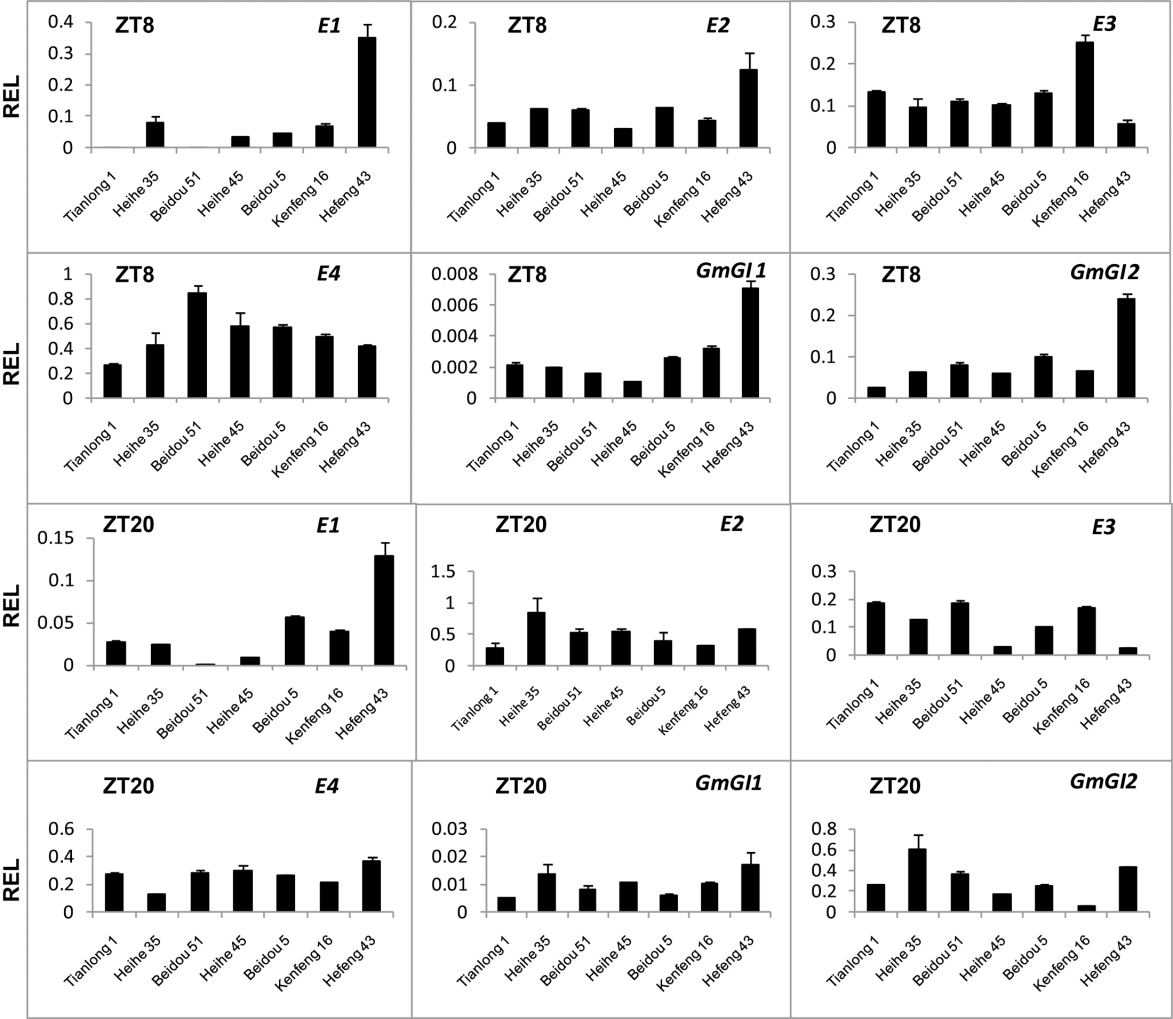
*E* gene expression levels in leaves of seven soybean cultivars. Relative expression levels (REL) were analyzed by RT-qPCR and normalized to *UKN1*. Averages and standard errors are the result of three replicates. For detailed information on stages and sampling, see Materials and Methods section.

To analyze the activity of *E1*, *E2*, *E3* and *E4* in soybean cultivars used in this study, we employed RT-qPCR to evaluate their expression in short day conditions. The results show that *E1*, *E2*, *E3*, *E4* and *E2* homeologs (*GmGI1* and *GmGI2*, [54]) expressed both in the morning (ZT8),and in the evening (ZT20) in all cultivars to different extents (Fig 2). Therefore, these *E* genes may not mutate and work normally in these cultivars.

### *GmFTL3* is most abundant among *GmFTL*s regardless of cultivars

**Fig 3:**
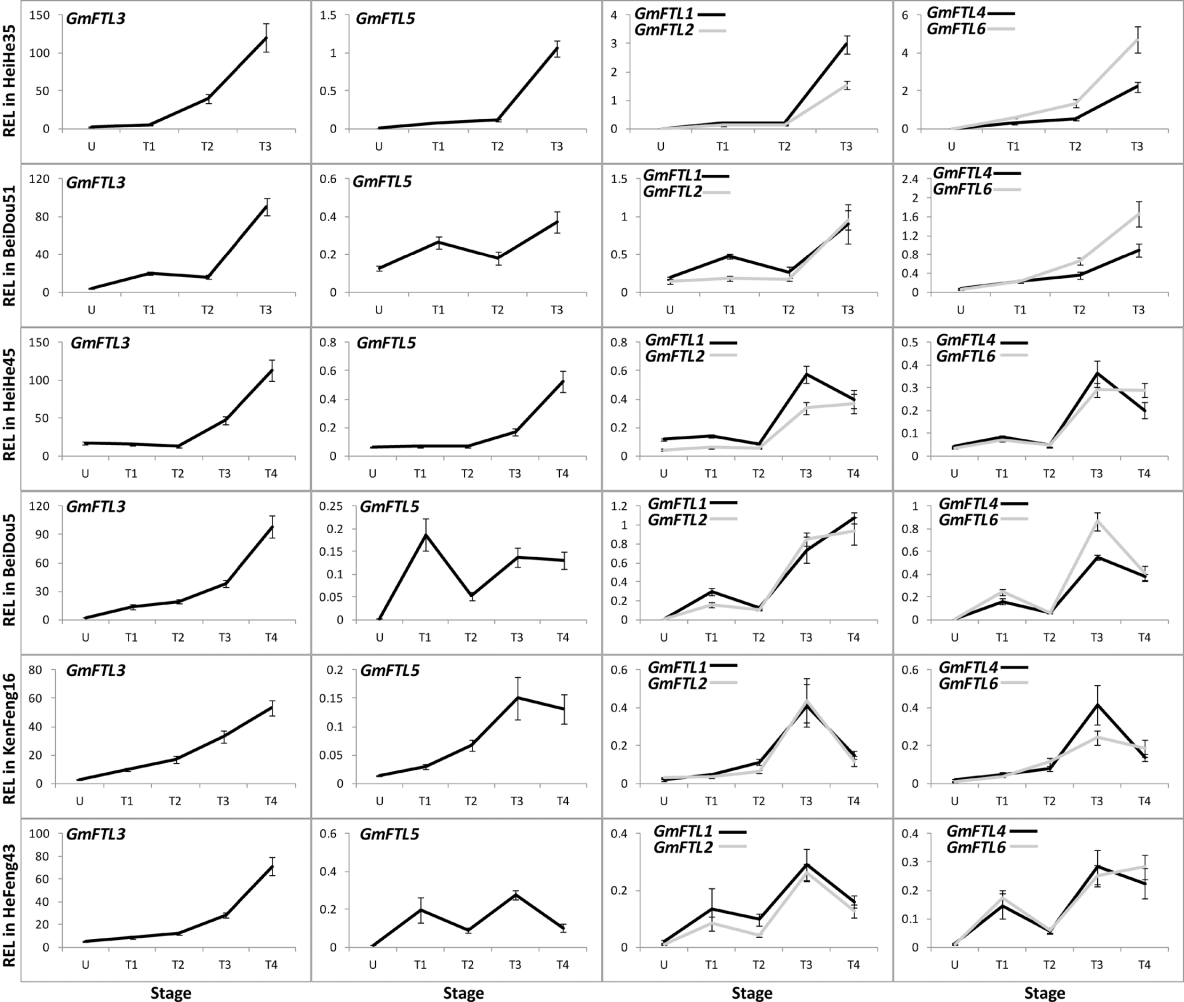
Relative expression levels of *GmFTL*s in six soybean cultivars at different stages. U, U-stage; T1, T1-stage; T2, T2-stage; T3, T3-stage; T4, T4-stage. Relative expression levels (REL) were analyzed by RT-qPCR and normalized to *UKN1*. Averages and standard errors are the result of three replicates. For detailed information on stages and sampling, see Materials and Methods section.

To understand the functional divergence of *GmFTL*s in flowering control, we carried out extensive analysis of expression patterns in different cultivars with different maturities (Table 1). The total transcripts of different *GmFTL*s in soybean increased along with plant growth, and reached peaks at the stage of the 3rd trifoliolates (T3 stage, Fig 3). Among them, *GmFTL3* exhibited both more or less identical patterns in all cultivars and the highest abundance among the *FT* homologs in all stages, suggesting that *GmFTL3* may play a most important role for flower induction in different cultivars. The expression of *GmFTL5*, the closest homeolog of *GmFTL3* (a pair-wise homeolog, with close relationship; [23]), did not show such a pattern among cultivars, indicating its expression divergences among cultivars. However, the other two pair-wise homeologs (*GmFTL1* and *GmFTL2* or *GmFTL4* and *GmFTL6*) displayed similar changes across plant growth in a given cultivar, even though some differences existed among different cultivars. Fig 3 also showed the phenomenon that early maturity cultivars (Heihe 35 and Beidou 51) had relatively higher levels of *GmFTL*s. The results imply that all *GmFTL*s might contribute to flowering induction in soybean cultivars, and higher levels of *GmFTL*s probably lead to early flowering and rapid maturity.

### All individual leaves express *GmFTL*s

**Fig 4:**
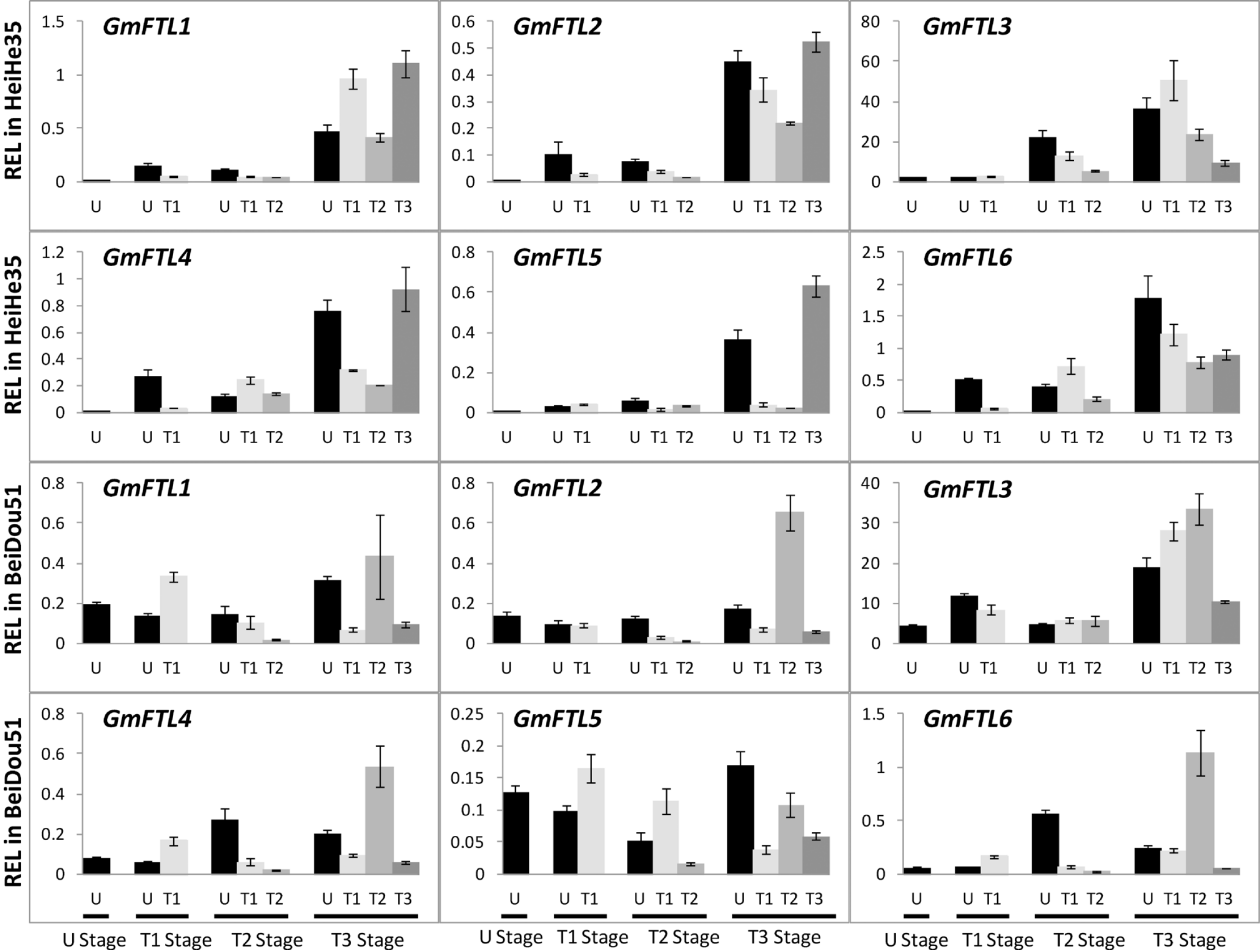
Relative expression level of *GmFTL*s in different leaves in cultivars Heihe 35 and Beidou 51 at different stages. Leaves: U, unifoliolate; T1, the 1^st^ trifoliolate; T2, the 2^nd^ trifoliolate; T3, the 3^rd^ trifoliolate stage. Relative expression levels (REL) were analyzed by RT-qPCR and normalized to *UKN1*. Averages and standard errors are the result of three replicates. For detailed information on stages and sampling, see Materials and Methods section.

Next, we analyzed the contribution of different leaves to production of *GmFTL*s for flowering control. Therefore, we analyzed the patterns of all *GmFTL*s in different leaves (Fig 4). As expected, the expression of a given *GmFTL* was not restricted to specific leaves, but detectable in all leaves. However, the abundance of different *GmFTL*s varied in different leaves and also had no consistency among cultivars. For example, in Heihe 35, unifoliolates and the first and third trifoliolates had relatively high level of *GmFTL*s, while the second trifoliolates produced less *GmFTL*s; but for Beidou 51, it appeared to be the opposite, especially when plants were flowering (T3 stage). The results indicate that all leaves contribute to flowering control, but to different extents in a cultivar-dependent mode.

### *GmFTL*s show a developmentally regulated expression

In early maturity cultivars (Heihe 35 and Beidou 51), expression of different *GmFTL*s in various leaves appeared to increase with developmental progress (Fig 3). In comparison with expression levels in a given leaf in early maturity cultivars, three pair-wise homeologous *GmFTL* genes (*GmFTL3* vs. *GmFTL5*, *GmFTL1* vs. *GmFTL2*, or *GmFTL4* vs. *GmFTL6*) exhibited expression divergence, especially after plants emerged flowers. In case of middle (Heihe 45 and Beidou 5, S1 Fig) and late (Kenfeng 16 and Hefeng 43, S2 Fig) maturity cultivars, there were similar patterns, even though the relationship between *GmFTL* expression and both flowering time and maturity are less stringent than that in early maturity cultivars.

### *GmFTL*s exhibit a divergence of circadian expression

**Fig 5:**
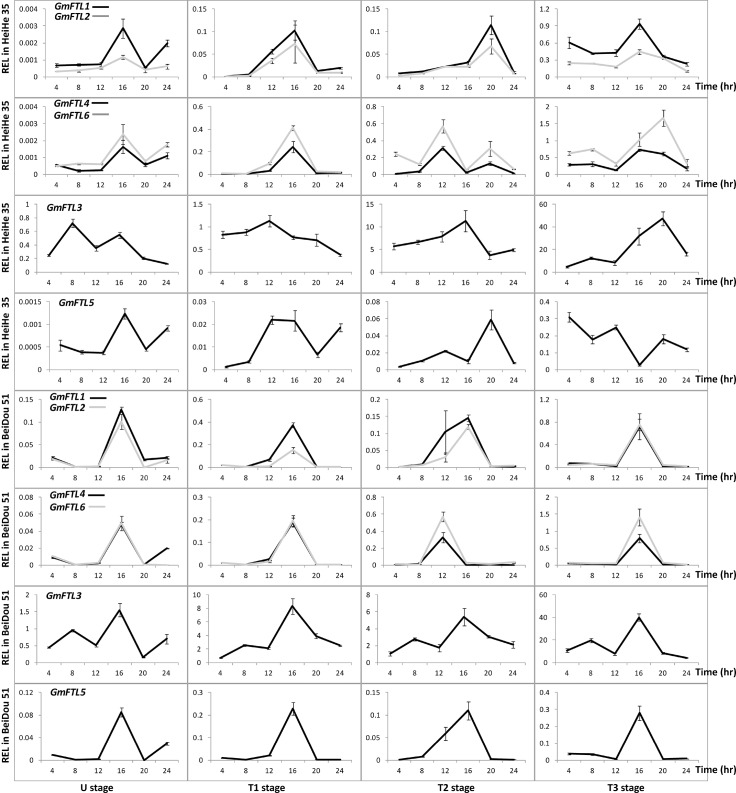
The diurnal rhythm of *GmFTL* expression in cultivars Heihe 35 and Beidou 51 at different stages. Relative expression levels (REL) were analyzed by RT-qPCR and normalized to *UKN1*. Averages and standard errors are the result of three replicates. For detailed information on stages and sampling, see Materials and Methods section.

Circadian expression is a typical character of florigen gene *FT*. A given *GmFTL* expressed in similar diurnal patterns, even though different patterns prevailed among different *GmFTL*s (Fig 5), indicating robust, but different circadian patterns. Pair-wise *GmFTL*s (*GmFTL1* and *GmFTL2*, *GmFTL4* and *GmFTL6*) also showed similar diurnal patterns, whereas the patterns of *GmFTL3* and *GmFTL5* were quite divergent (Fig 5, S3 and S4 Figs). In *Arabidopsis*, the *FT* expressing peak occurs at dusk [55]. In early stages, there were two peaks of *FT* transcripts, one in the morning (at 8:00), and the other in the afternoon (at 16:00), respectively (Fig 5, S3 and S4 Figs). The results show that different diurnal-regulation mechanisms exist for *FT* homeologs between soybean and *Arabidopsis*.

### *GmCOL1*, *GmCOL2*, *GmCOL5*, and *GmCOL13* may all contribute to *GmFTL* expression

**Fig 6:**
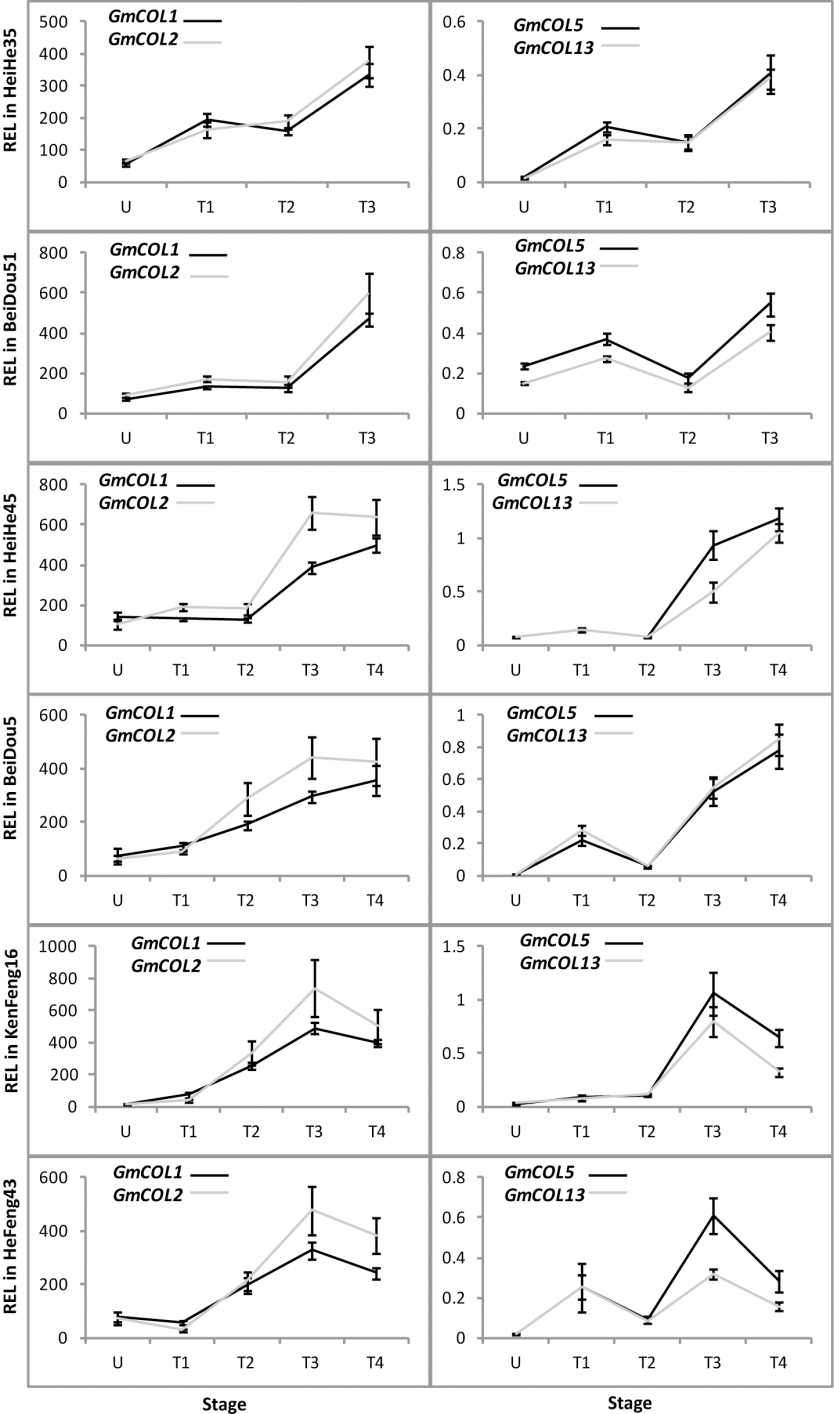
Relative expression level analyses of *GmCOL*s in six soybean cultivars at different stages. Relative expression levels (REL) were analyzed by RT-qPCR and normalized to *UKN1*. Averages and standard errors are the result of three replicates. For detailed information on stages and sampling, see Materials and Methods section.

**Fig 7:**
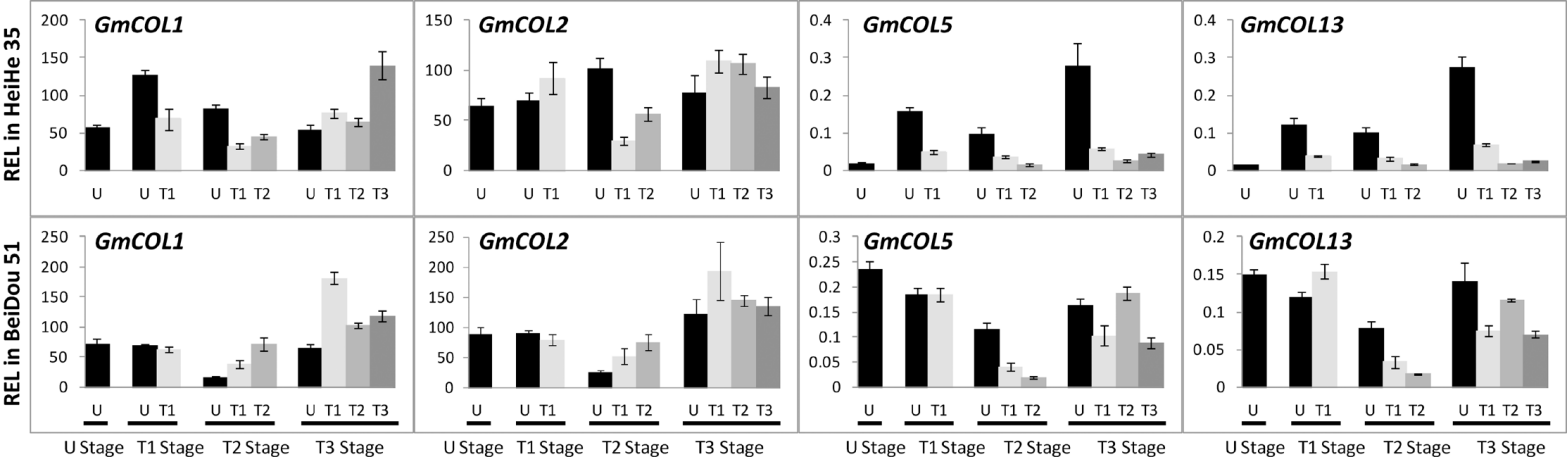
Relative expression level analyses of *GmCOL*s in different leaves in cultivars Heihe 35 and Beidou 51 at different stages. Relative expression levels (REL) were analyzed by RT-qPCR and normalized to *UKN1*. Averages and standard errors are the result of three replicates. For detailed information on stages and sampling, see Materials and Methods section.

**Fig 8:**
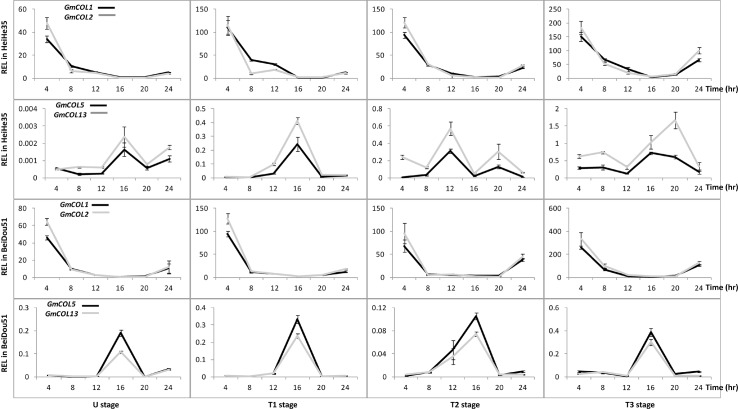
The diurnal rhythm of *GmCOL* expression in cultivars Heihe 35 and Beidou 51 at different stages. Relative expression levels (REL) were analyzed by RT-qPCR and normalized to *UKN1*. Averages and standard errors are the result of three replicates. For detailed information on stages and sampling, see Materials and Methods section.

In various plants, the *FT* gene is regulated by multiple genes, and *CONSTANS* (*CO*) is the main up-regulator of *FT* in the photoperiodic pathway [1, 7]. Soybean also has multiple copies of *CO* homologs [23]. So the question is that which one of the *CO*-*like* genes regulates a specific *FT* homolog for flowering in soybean. To evaluate it, we further investigated the expression pattern of *GmCOL*s with the same set of samples used for *GmFTL* expression. Based on our previous studies [23], we focused on four *CO* homolog candidates (*GmCOL1* and *GmCOL2*, *GmCOL5*, and *GmCOL13*) in soybean cultivars Heihe 35 and Beidou 51. As expected, *GmCOL1* and *GmCOL2* or *GmCOL5* and *GmCOL13* had patterns similar to each other in developmental (Fig 6), tissue-/organ- (Fig 7), and circadian (Fig 8) regulation as the *GmFTL*s. The level of *GmCOL1* and *GmCOL2* were obviously higher than that of *GmCOL5* and *GmCOL13*. In case of the circadian patterns, *GmCOL1* and *GmCOL2* peaked in the morning, while the highest level of *GmCOL5* and *GmCOL13* appeared in the afternoon. This characteristic mirrored the two peaks of *GmFTL*s, respectively (Fig 5). Comparison of *GmCOL* expression among different cultivars showed similar modes (S5–S8 Figs). The results suggest that individual *GmCOL*s do not regulate one specific *GmFTL*, but morning *GmCOL1/2* may be related to the morning peak of *GmFTL*s, while *GmCOL5/13* contribute the afternoon peak of *GmFTL*s.

### Ectopic expression of *GmFTL4* results in early flowering in soybean

**Fig 9:**
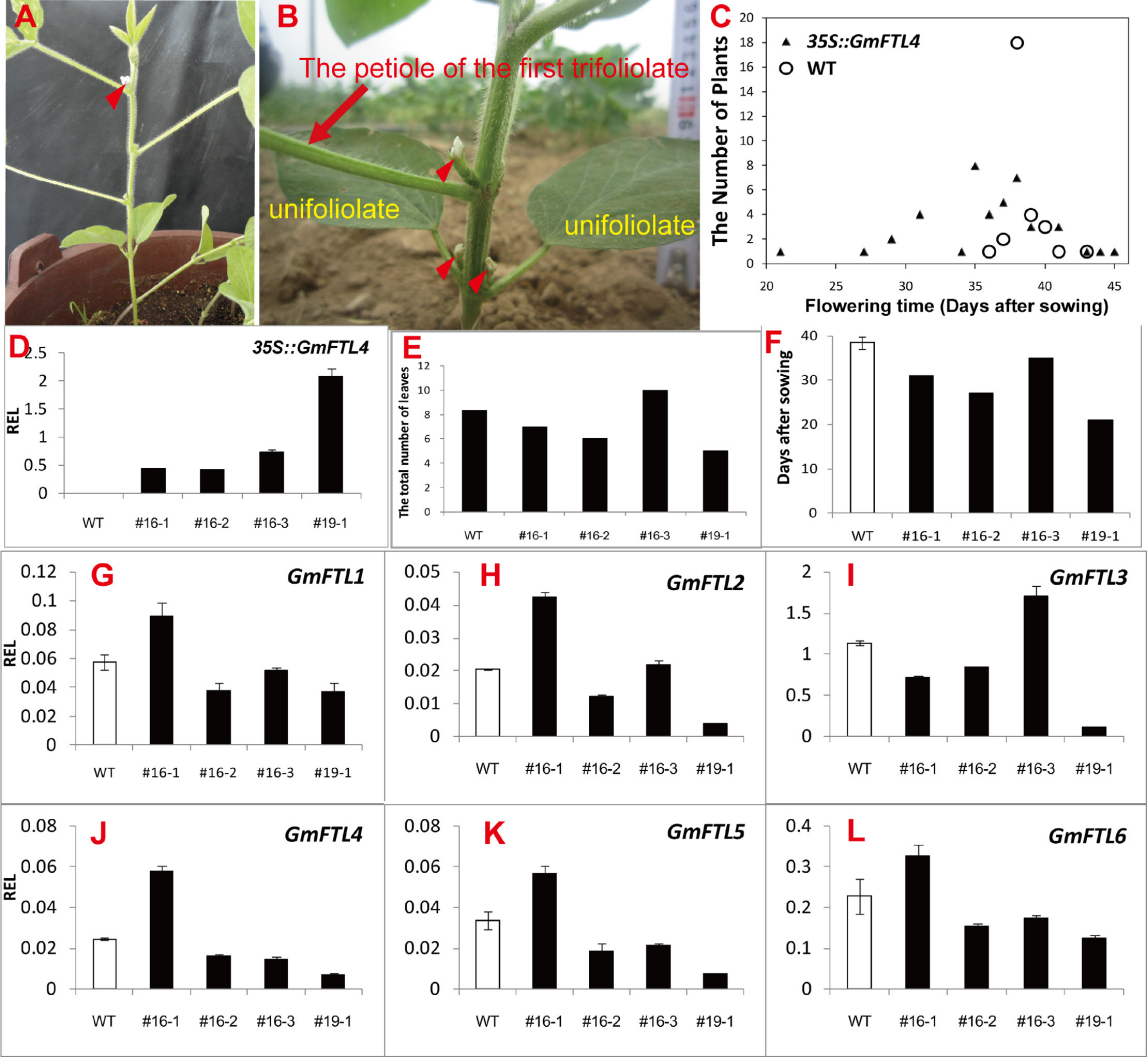
Overexpression of *GmFTL4* causes precocious flowering in the soybean cultivar Tianlong 1. (A) A wild-type Tianlong 1 plant showing the first flower appearing at the 3^rd^ trifoliolate axil (red arrow-heads). (B) A transgenic line indicating flowers (red arrow-heads) appearing at the unifoliate (yellow words) and the first trifoliolate axil (red arrow and words indicating the petiole of the first trifoliolate). (C) The flowering times of different transgenic lines compared with wild-type plants (WT). (D) Relative expression level (REL) analyses of transgene-derived *GmFTL4* in transgenic lines and wild-type plants (WT). The total number of leaves at flowering and flowering times of the transgenic plants were shown in (E) and (F), respectively. (G) to (L) Relative expression level analyses of endogenous *GmFTLs* in transgenic lines in field conditions. Trifoliolates were sampled at 40 days after sowing.

We expressed *GmFTL4-GFP* driven by the 35S promoter in cultivar Tianlong 1, a cultivar from central China (location: EL113°41′- 115°05′, NL28°58′- 31°22′). We got 20 independent transgenic lines in total. Most of the lines (62%) showed early flowering phenotypes with the shortest flowering time of 21 days after germination (Fig 9C) in the Zhengzhou area (location: EL112°42′- 114°14′, NL34°16′- 34°58′) (S9 Fig). The first flowers appeared in unifoliolate axils in transgenic lines (Fig 9B), except one line (#16–3), where they appeared in the first, third or fourth trifoliolate axils. In comparison, flowers in the parent line appeared in the third or fourth axils (Fig 9A). Four lines were selected for further analysis of gene expression. Data from RT-qPCR (Fig 9D) clearly displayed that transgenic *GmFTL4* over-expressed in the lines, and their levels were corresponding to flowering phenotypes, that is, the higher the level, the earlier flowering (Fig 9D and 9E). We then investigated the effect of exogenous *GmFTL4* on the expression of endogenous *GmFTL*s. The results showed the fluctuation of different *GmFTL*s’ expression was observed to various extents compared to that in WT soybean (Fig 9G–9L): in three lines (#16–2, #16–3, and #19–1) the expression of *GmFTL1*, *2*, *4*, *5*, and *6* was dampened, while in line #16–1 the expression of these genes was enhanced. The change of *GmFTL3* expression was interesting: enhanced in line #16–3, repressed in other lines. In our opinion, these effects may possibly result from different insertion positions of T-DNA and/or interfering/co-suppression between exogenous and endogenous *GmFTL* genes. The phenotype of flowering times was a complex result of interaction between exogenous and endogenous genes. Another obvious and interesting character was the relationship between *GmFTL3* and the position of the first flowers. Higher *GmFTL3* (Line #16–3) resulted in flowers appearing in trifoliolate axils and lower *GmFTL3* (Line #16–1, #16–2, and #19–1) resulted in flowers appearing in unifoliolate axils, indicating a role of *GmFTL3* in shoot growth.

### Knock-down of *GmFTL* expression delays flowering in soybean

**Fig 10:**
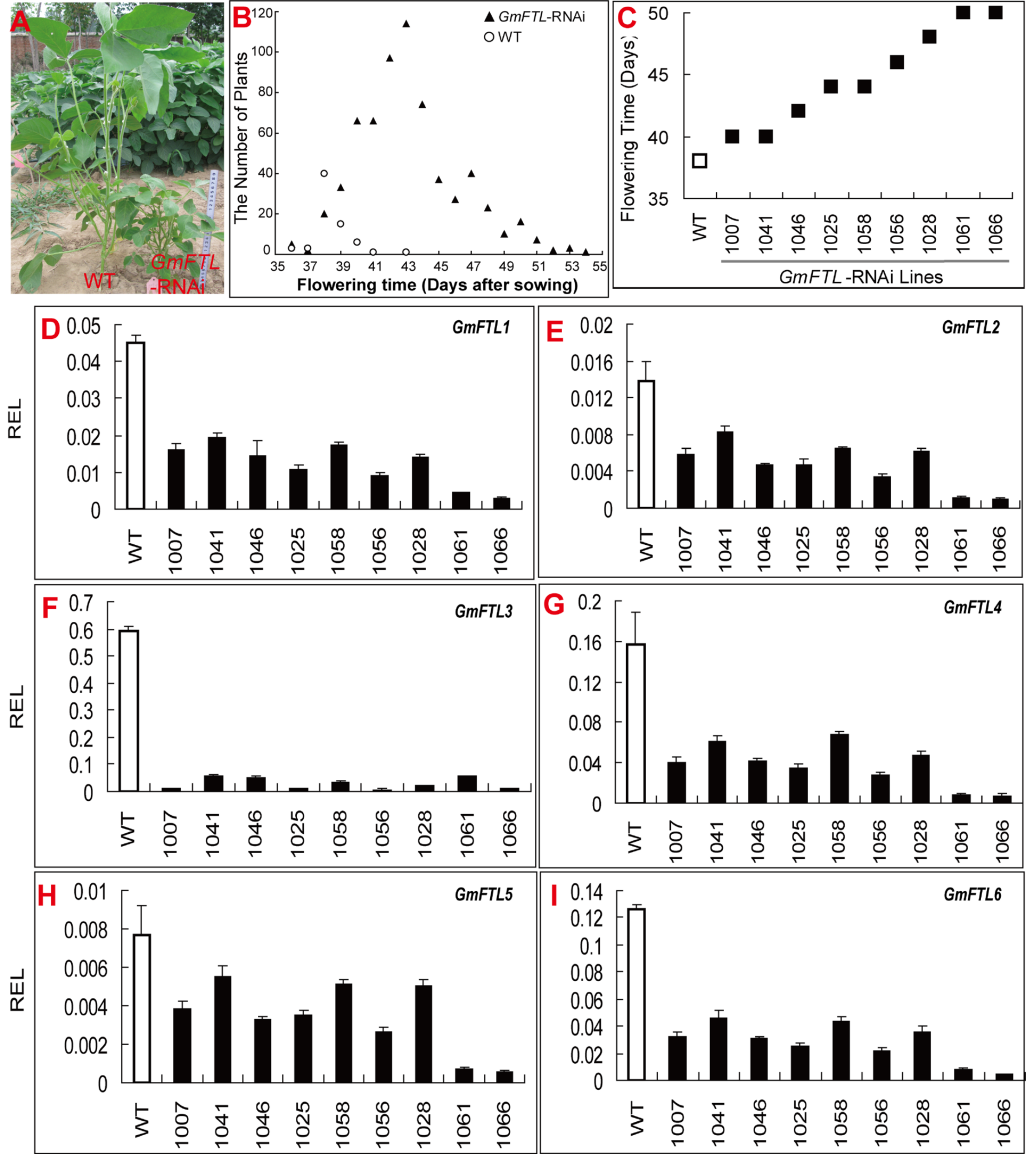
Silencing *GmFTL*s delays flowering and inhibits growth. (A) A *GmFTL*-RNAi transgenic (right) and wild type (left) soybean plants grown in the field for 40 days. (B) Flowering time of transgenic lines. The total number of plants: WT, 69; Transgenic lines, 643. (C) The flowering time of transgenic lines used for analysis of *GmFTL* relative expression level (REL, D to I). (D to I) Endogenous *GmFTL* relative expression level in *GmFTL* silencing lines. Trifoliolates were sampled at 40 days after sowing and leaves from five plants of each line were mixed for RT-qPCR (C to I). Data are mean +/- SD from three technical replicates.

To further investigate the function of *GmFTL*s, RNA silencing approach was employed. Due to highly conserved sequences among *GmFTL*s, an RNAi-fragment based on *GmFTL1* coding-sequence (cds) was used as the target sequence, which was expected to target all *GmFTL*s (S10A Fig). Sixteen independent lines were generated. Most lines displayed late flowering phenotype with the latest one of 50 days after sowing compared with 38 days for WT (Fig 10A and 10B). We grouped these lines according to their flowering times in 2 day intervals (Fig 10C), and selected nine lines from different groups to check the silencing efficiency. RT-qPCR data indicated that all endogenous *GmFTL*s were knocked down in all lines with the largest effect on *GmFTL3* (95.6%, S10B Fig). The efficiency of knock-down was also consistent with the extent of flowering time delay (Fig 10C–10I and S10B Fig), suggesting that all of *GmFTL*s may contribute to flowering control. However, the relative level of *GmFTL3* was still higher than that of other *GmFTL*s (Fig 10D–10I), and these transgenic lines had no flowers in unifoliolate axils, further inferring that *GmFTL3* functioned in determination of the flower position on the stem.

Another significant phenotype was that all *GmFTL*-RNAi plants had shorter/smaller stature than WT (Fig 10A). Generally, knock-down or knock-out of flowering enhancers result in a higher/larger stature phenotype in *Arabidopsis* [55] and rice [13]. Therefore, the dwarf phenotype inferred additional and different functions of *GmFTL*s in soybean.

## Discussion

Accumulating evidences support that *FT*, a highly conserved florigen gene across plants, occurs in many plants in multiple copies, which display functional variation [7, 56]. In soybean, there are at least six *GmFTL*s showing flowering activity [23, 24, 26–28]. However, the functional divergence of *GmFTL*s that occurred in soybean cultivars is unknown. In this study, we focus on analysis of *GmFTL* expression spectra in different cultivars under field conditions, and show that *GmFTL*s’ expression correlates with cultivar maturity, in that early maturity cultivars have higher levels of *GmFTL*s. And knockdown of *GmFTL*s delay flowering. Such a conclusion is in accordance with what happens in other species, including *Arabidopsis*, rice, wheat (review in [1, 57–59]). This suggests that all of *GmFTL*s impact on flowering induction and domestication-related diversification of soybean. In comparison with expression levels in a given leaf in early maturity cultivars, three pair-wise homeologous *GmFTL* genes (*GmFTL3* vs. *GmFTL5*, *GmFTL1* vs. *GmFTL2*, or *GmFTL4* vs. *GmFTL6*) exhibit expression divergence, especially after plants emerge flowers. In case of diurnal expressions, different *GmFTL*s also show various patterns, even the patterns of *GmFTL3* and *GmFTL5* (pair-wise genes) are quite divergent. Such expression differentiation may contribute to survival of duplicated genes [60] after genome duplication events during a long-term evolutional progress [61, 62]. The expression pattern of *GmFTL3* in different soybean cultivars is quite similar and highly conserved, but other *GmFTL*s have cultivar-specific patterns with relatively low levels, indicating that with progress of domestication, soybean *GmFTL3* retained the conserved function in flowering regulation, while *GmFTL1/2/4/5/6* underwent divergence to adapt to different environments.

Even though different copies of *GmFTL*s display different expression levels, *GmFTL3* (*GmFT2a*) shows the highest abundance among them, regardless of developmental-, organ-, and circadian-specific expressions and cultivars, indicating that *GmFTL3* may be the major florigen, that is consistent with previous report [24, 26]. However, the function of other *GmFTL*s on the regulation of flowering time can’t be ignored, because exogenous *GmFTL*s expressed from the 35S promoter enhance flowering in soybean or *Arabidopsis* (this study, [23, 24, 26–28], and the pattern of developmental and circadian expressions is still similar among *GmFTL*s. To analyze flowering phenotype of *GmFTL* mutants and to express *GmFTL*s from their native promoters in soybean will help to understand their contribution to florigen.

*GmFTL3* not only has high abundance, but also a unique circadian pattern,with two peaks in the early stage, one in morning and the other one in the afternoon. Other *GmFTL*s have their unique peak expressions in the morning or afternoon. On the other way, the transcript level of both *GmCOL1/2* peaks in the morning, while that of *GmCOL5*/*13* peaks in the afternoon. We speculate that there may be at least two *CO*-*FT* regulons in soybean flowering network, one includes *GmCOL1*/*2* and morning-phase *GmFTL*s, the other contains *GmCOL5*/*13* and afternoon-/evening-phase *GmFTL*s. These two *CO*-*FT* regulons may coordinately control flowering time in soybean. Certainly, more lines of evidence are needed to support this speculation.

Many studies in *Arabidopsis* used whole seedlings, which include many leaves and roots, as materials for investigation of flowering induction. However, there is no evidence showing a differential influence of different leaves on flowering regulation, even though most of *Arabidopsis* wild types have at least 10 leaves before flowering. Recent evidences show that cotyledons alone are enough for *Arabidopsis* floral induction [63]. In *Pharbitis nil* [64] and pea [65], cotyledons are also involved in flower initiation. Soybean plants produce several (3–4 for cultivars used here) trifoliolate leaves before the first flower emerges. However, our previous research indicated that unifoliolates are sufficient to induce soybean flowering [38]. To sum up our data of expression analysis, all leaves express different *GmFTL*s. Therefore, all leaves may contribute to floral induction.

Previous papers reported that maturity loci *E1*, *E2*, *E3* and *E4* have important, but different impacts on maturity and photoperiod response, and they also indicate that it is unique in photoperiod sensitivity for different cultivars and there are other genes involved in photoperiod response [66–68]. In this study, we did not found parallel relationship between *E1* expression and both *GmFTL/GmCOL* expression and cultivars maturity for these six soybean cultivars. *E1* abundance of transcripts displays an opposite relationship to cultivar maturity except cultivars Heihe 35 and Tianlong 1, similar to previous reports [67, 69]. A similar situation was found for *E2*, *E3* and *E4*. This information may indicate much complicated mechanisms of *GmFTL* regulation happened in different cultivars.

Expression data also show that *GmFTL3* is the gene expressed at the highest level gene in different cultivars, and the first flowers appears in the trifoliolate axils of WT plants. Overexpression of *GmFTL*s results in formation of flowers in unifoliolate axils (this study and [26]). In overexpressing transgenic line #16–3, there are no flowers in unifoliolate axils even though exogenous *GmFTL4* is overexpressed. Detailed comparison of expression levels of *GmFTL*s in different transgenic lines reveals that the expression balance between *GmFTL3* and other *GmFTL*s in plants contributes to the position of the first flowers.

*GmFTL*s may also participate the regulation of plant vegetative growth. In *Arabidopsis* [55] and rice [13], *FT* expression enhances flowering and inhibits vegetative growth. Therefore, knock-down or mutation of the *FT* gene results in late flowering and higher and larger stature of plants. However, all knock-down transgenic lines of soybean in this study have much shorter stature than their parents, suggesting that *GmFTL*s are involved in stem growth in soybean. An alternative explanation is that *GmFTL*s may control both reproduction and vegetative growth in a *GmFTL*-species dependent mode.

Introduction of elite soybean varieties into different latitude areas is important but challenging, because soybean is a qualitative/absolute short-day plant [70], whose flowering is highly sensitivity to day length. Therefore, photoperiod sensitivity is a key factor, which limits elite introduction between different areas. We show that overexpression or silencing of *GmFTL*s can significantly change flowering time. Therefore, it is possible to design soybean plants with a desired flowering time by controlling the expression level of *GmFTL*s. For instance, plant breeders can introduce transgenic lines with special flowering times, as shown in this study, into a soybean elite variety of interest to modulate its flowering time. Of course, the effect of modulation of *GmFTL* expression on other agronomic traits, such as yield and resistance to biotic/abiotic stress, should be carefully evaluated.

## Materials and Methods

### Plant Materials

Six soybean cultivars are from the northeastern area, Heilongjian province of China as Table 1 showed. All seeds from Dr. Qingshan Chen lab at College of Agriculture, Northeast Agricultural University. These cultivars were grown in pots for sampling, or in the same field with the same cultivation management for phenotype assessment and seed reproduction in Harbin, Heilongjiang Province, China (Fig 1). For sampling, six seeds from one pot were sown on May 11, 2013, and harvested on Aug.18, 2013 (for Heihe 35 and Beidou 51), or on Sept. 13, 2013 (for Heihe 45, Beidou 5, Kenfeng 16 and Hefeng 43). The samples of leaves for RT-qPCR analysis were harvested on July, 1/7/12/18, 2013, respectively. For phenotype and seed reproduction, the seeds of all cultivars were sown on Apr. 25, 2013, and harvested on Sept. 20, 2013. The unifoliolate and different trifoliolate leaves were sampled at different stages before flowering. Stages were defined as the time when the unifoliolates or trifoliolates expanded fully, therefore we harvested samples at unifoliolate stage (U-stage), the 1^st^ trifoliolate stage (T1-stage), the 2^nd^ trifoliolate stage (T2-stage), the 3^rd^ trifoliolate stage (T3-stage), and the 4^th^ trifoliolate stage (T4-stage). For developmental samples, different leaves were combined at a given stage. For circadian samples, leaves were collected at 4 hour intervals at different stages (S2 Table). All the samples above were combined with at least five individual plants. For transgenic plants, five middle-leaflets of the 3^rd^ trifoliolates (counting from the top) from five individual plants were combined as samples at day 40 after sowing. Three groups of samples from different pods were harvested for the parallel analysis. All samples were immediately frozen in liquid nitrogen and stored at -80°C until used.

Transgenic plants were grown in the field of Zhengzhou, China, which locates at EL112°42'- 114°14' and NL34°16'- 34°58', and is little higher latitude than Wuhan, China (EL113°41′- 115°05′, NL28°58′- 31°22′), a city where its WT cultivar Tianlong 1 originated from. The daylength change during the growth season is shown in S9 Fig The seeds were sown on June 10, 2014, and harvested on Oct 12, 2014. The samples of leaves for RT-qPCR analysis were harvested on July 18, 2014.

For expression evaluation of *E* genes, 7 cultivars were grown in a growth chamber under short day conditions (8 hrs/16 hrs, light and dark; light intensity: 270 μmol·m^-1^·s^-1^). Leaf samples of the 1^st^ trifoliolates at the 1^st^ trifoliolate stage (T1-stage) were harvested at Zeitgeber (ZT) 8 and ZT20 for RT-qPCR analysis.

### RNA isolation, cDNA synthesis and RT-qPCR analysis

Total RNA was extracted from various leaves using TRIzol (Invitrogen, USA). The RNA was treated with RNase-free recombinant DNase I (Takara, Dalian, China). The integrity of the RNA was checked electrophoretically and quality assessment of total RNA was checked with NanoDrop ND-2000 Spectrophotometer (Thermo Scientific, Wilmington, DE, USA). The isolated RNA was then subjected to reverse transcription using the Super-Script III Reverse Transcriptase kit. Real-time quantitative PCR (RT-qPCR) was performed with the SYBR Green Mix (Takara, Dalian, China) on StepOnePlus Real-Time PCR System (Applied Biosystems, USA) according to the manufacturer’s protocol. *GmUKN1* (Glyma12g02310) was selected as the reference gene. Three independent biological replicates were performed. Raw data were standardized as described previously [71]. All primers are listed in S3 Table.

### Construction of plasmids

The full-length CDS of *GmFTL4* was amplified from our previous clone [23] using the primer pairs of *GmFTL4*-*Asc* I-5941-F and *GmFTL4*-*Xba* I-R (S3 Table), and then inserted into the pFGC5941-GFP vector, which was constructed by inserting the GFP gene into pFGC5941 (GenBank Accession No AY310901) between *Xba* I and *Sma* I sites, to obtain pFGC5941-*GmFTL4*-GFP as *GmFTL4-GFP* expressing construct. For *GmFTL*s silencing, a fragment of *GmFTL1* (S10 Fig) was cloned into pFGC5941 at *Nco* I / *Asc* I and *Avr* II / *Xba* I sites, to obtain binary vector pFGC5941-*GmFTL*. Therefore, both *GmFTL4* and *GmFTL1*-silencing fragments were driven by the cauliflower mosaic virus 35S promoter.

### Soybean genetic transformation

The *Agrobacterium* strain EHA105 containing constructs pFGC5941-*GmFTL4*-GFP or pB7G-*GmFTL* was used to transform the soybean cultivar Tianlong 1, following the cotyledonary node method [72–74]. Transformants were screened by applying160 mg/L glufosinate onto the preliminary leaves of the seedlings. Transgenic plants in the greenhouse and field were sprayed with 1/1000 herbicide Basta. The herbicide-resistant plants were subjected to molecular and phenotypic analysis. For phenotype (flowering and stature) analysis, T2 and T3 lines (for RNAi transgenic plants) or T1 (for overexpressing transgenic plants) were used.

## Supporting Information

S1 Dataset (PDF)

S1 Fig Tissue-specific expression patterns of *GmFTL*s in soybean cultivars HeiHe 45 and BeiDou 5. (PDF)

S2 Fig Tissue-specific expression patterns of *GmFTL*s in soybean cultivars Kenfeng 16 and Hefeng 43. (PDF)

S3 Fig Circadian expression patterns of *GmFTL*s in soybean cultivars HeiHe 45 and BeiDou 5. (PDF)

S4 Fig Circadian expression patterns of *GmFTL*s in soybean cultivars Kenfeng 16 and Hefeng 43. (PDF)

S5 Fig Tissue-specific expression patterns of *GmCOL*s in soybean cultivars HeiHe 45 and BeiDou 5. (PDF)

S6 Fig Tissue-specific expression patterns of *GmCOL*s in soybean cultivars Kenfeng 16 and Hefeng 43. (PDF)

S7 Fig Circadian expression patterns of *GmCOL*s in soybean cultivars HeiHe 45 and BeiDou 5. (PDF)

S8 Fig Circadian expression patterns of *GmCOL*s in soybean cultivars Kenfeng 16 and Hefeng 43. (PDF)

S9 Fig The day length change in Zhengzhou. (PDF)

S10 Fig The strategy and efficiency of *GmFTL* silencing. (PDF)

S1 Table The names of *GmFTL*s in this study and literature. (PDF)

S2 Table Sampling organs and date/time for gene expression analysis of circadian. (PDF)

S3 Table The list of primers used in this study. (PDF)

## References

[1] Andres F, Coupland G. The genetic basis of flowering responses to seasonal cues. Nat Rev Genet. 2012;13(9):627–39. 10.1038/nrg3291 . 22898651

[2] Simpson GG. Evolution of flowering in response to day length: flipping the CONSTANS switch. Bioessays. 2003;25(9):829–32. . 12938171 10.1002/bies.10330

[3] Ballerini ES, Kramer EM. In the Light of Evolution: A Reevaluation of Conservation in the CO-FT Regulon and Its Role in Photoperiodic Regulation of Flowering Time. Frontiers in plant science. 2011;2:81 10.3389/fpls.2011.00081 22639612 PMC3355682

[4] Bohlenius H, Huang T, Charbonnel-Campaa L, Brunner AM, Jansson S, Strauss SH, et al CO/FT regulatory module controls timing of flowering and seasonal growth cessation in trees. Science. 2006;312(5776):1040–3. . 16675663 10.1126/science.1126038

[5] Karlgren A, Gyllenstrand N, Kallman T, Sundstrom JF, Moore D, Lascoux M, et al Evolution of the PEBP gene family in plants: functional diversification in seed plant evolution. Plant Physiol. 2011;156(4):1967–77. 10.1104/pp.111.176206 21642442 PMC3149940

[6] Chardon F, Damerval C. Phylogenomic analysis of the PEBP gene family in cereals. J Mol Evol. 2005;61(5):579–90. 10.1007/s00239-004-0179-4 . 16170456

[7] Pin PA, Nilsson O. The multifaceted roles of FLOWERING LOCUS T in plant development. Plant Cell Environ. 2012;35(10):1742–55. 10.1111/j.1365-3040.2012.02558.x . 22697796

[8] Corbesier L, Vincent C, Jang S, Fornara F, Fan Q, Searle I, et al FT protein movement contributes to long-distance signaling in floral induction of Arabidopsis. Science. 2007;316(5827):1030–3. . 17446353 10.1126/science.1141752

[9] D'Aloia M, Bonhomme D, Bouche F, Tamseddak K, Ormenese S, Torti S, et al Cytokinin promotes flowering of Arabidopsis via transcriptional activation of the FT paralogue TSF. Plant J. 2011;65(6):972–9. 10.1111/j.1365-313X.2011.04482.x . 21205031

[10] Hiraoka K, Yamaguchi A, Abe M, Araki T. The florigen genes FT and TSF modulate lateral shoot outgrowth in Arabidopsis thaliana. Plant Cell Physiol. 2013;54(3):352–68. 10.1093/pcp/pcs168 . 23220822

[11] Tamaki S, Matsuo S, Wong HL, Yokoi S, Shimamoto K. Hd3a protein is a mobile flowering signal in rice. Science. 2007;316(5827):1033–6. . 17446351 10.1126/science.1141753

[12] Komiya R, Yokoi S, Shimamoto K. A gene network for long-day flowering activates RFT1 encoding a mobile flowering signal in rice. Development. 2009;136(20):3443–50. 10.1242/dev.040170 19762423

[13] Komiya R, Ikegami A, Tamaki S, Yokoi S, Shimamoto K. Hd3a and RFT1 are essential for flowering in rice. Development. 2008;135(4):767–74. 10.1242/dev.008631 . 18223202

[14] Meng X, Muszynski MG, Danilevskaya ON. The FT-Like ZCN8 Gene Functions as a Floral Activator and Is Involved in Photoperiod Sensitivity in Maize. The Plant cell. 2011 Epub 2011/03/29. tpc.110.081406 [pii] 10.1105/tpc.110.081406 . 21441432 PMC3082274

[15] Navarro C, Abelenda JA, Cruz-Oro E, Cuellar CA, Tamaki S, Silva J, et al Control of flowering and storage organ formation in potato by FLOWERING LOCUS T. Nature. 2011;478(7367):119–22. 10.1038/nature10431 . 21947007

[16] Hsu CY, Adams JP, Kim H, No K, Ma C, Strauss SH, et al FLOWERING LOCUS T duplication coordinates reproductive and vegetative growth in perennial poplar. Proc Natl Acad Sci U S A. 2011;108(26):10756–61. 10.1073/pnas.1104713108 21653885 PMC3127867

[17] Lifschitz E, Eviatar T, Rozman A, Shalit A, Goldshmidt A, Amsellem Z, et al The tomato FT ortholog triggers systemic signals that regulate growth and flowering and substitute for diverse environmental stimuli. Proc Natl Acad Sci U S A. 2006;103(16):6398–403. 10.1073/pnas.0601620103 16606827 PMC1458889

[18] Krieger U, Lippman ZB, Zamir D. The flowering gene SINGLE FLOWER TRUSS drives heterosis for yield in tomato. Nat Genet. 2010;42(5):459–63. 10.1038/ng.550 . 20348958

[19] Pin PA, Benlloch R, Bonnet D, Wremerth-Weich E, Kraft T, Gielen JJ, et al An antagonistic pair of FT homologs mediates the control of flowering time in sugar beet. Science. 2010;330(6009):1397–400. 10.1126/science.1197004 . 21127254

[20] Kinoshita T, Ono N, Hayashi Y, Morimoto S, Nakamura S, Soda M, et al FLOWERING LOCUS T regulates stomatal opening. Curr Biol. 2011;21(14):1232–8. 10.1016/j.cub.2011.06.025 . 21737277

[21] Xi W, Yu H. An expanding list: another flowering time gene, FLOWERING LOCUS T, regulates flower development. Plant Signal Behav. 2009;4(12):1142–4. 20514229 10.4161/psb.4.12.9901 PMC2819439

[22] Liu L, Farrona S, Klemme S, Turck FK. Post-fertilization expression of FLOWERING LOCUS T suppresses reproductive reversion. Frontiers in plant science. 2014;5:164 10.3389/fpls.2014.00164 24817870 PMC4012189

[23] Fan C, Hu R, Zhang X, Wang X, Zhang W, Zhang Q, et al Conserved CO-FT regulons contribute to the photoperiod flowering control in soybean. BMC Plant Biol. 2014;14:9 10.1186/1471-2229-14-9 24397545 PMC3890618

[24] Kong F, Liu B, Xia Z, Sato S, Kim BM, Watanabe S, et al Two coordinately regulated homologs of FLOWERING LOCUS T are involved in the control of photoperiodic flowering in soybean. Plant Physiol. 2010;154(3):1220–31. 10.1104/pp.110.160796 20864544 PMC2971601

[25] Wang Z, Zhou Z, Liu Y, Liu T, Li Q, Ji Y, et al Functional evolution of phosphatidylethanolamine binding proteins in soybean and Arabidopsis. The Plant cell. 2015;27(2):323–36. 10.1105/tpc.114.135103 . 25663621 PMC4456927

[26] Sun H, Jia Z, Cao D, Jiang B, Wu C, Hou W, et al GmFT2a, a soybean homolog of FLOWERING LOCUS T, is involved in flowering transition and maintenance. PloS one. 2011;6(12):e29238 10.1371/journal.pone.0029238 22195028 PMC3237611

[27] Nan H, Cao D, Zhang D, Li Y, Lu S, Tang L, et al GmFT2a and GmFT5a redundantly and differentially regulate flowering through interaction with and upregulation of the bZIP transcription factor GmFDL19 in soybean. PloS one. 2014;9(5):e97669 10.1371/journal.pone.0097669 24845624 PMC4028237

[28] Jiang B, Yue Y, Gao Y, Ma L, Sun S, Wu C, et al GmFT2a polymorphism and maturity diversity in soybeans. PloS one. 2013;8(10):e77474 10.1371/journal.pone.0077474 24155962 PMC3796496

[29] Putterill J, Robson F, Lee K, Simon R, Coupland G. The CONSTANS gene of Arabidopsis promotes flowering and encodes a protein showing similarities to zinc finger transcription factors. Cell. 1995;80(6):847–57. . 7697715 10.1016/0092-8674(95)90288-0

[30] Griffiths S, Dunford RP, Coupland G, Laurie DA. The evolution of CONSTANS-like gene families in barley, rice, and Arabidopsis. Plant Physiol. 2003;131(4):1855–67. . 12692345 10.1104/pp.102.016188 PMC166942

[31] Serrano G, Herrera-Palau R, Romero JM, Serrano A, Coupland G, Valverde F. Chlamydomonas CONSTANS and the evolution of plant photoperiodic signaling. Curr Biol. 2009;19(5):359–68. 10.1016/j.cub.2009.01.044 19230666

[32] Lagercrantz U, Axelsson T. Rapid evolution of the family of CONSTANS LIKE genes in plants. Mol Biol Evol. 2000;17(10):1499–507. . 11018156 10.1093/oxfordjournals.molbev.a026249

[33] Yano M, Katayose Y, Ashikari M, Yamanouchi U, Monna L, Fuse T, et al Hd1, a major photoperiod sensitivity quantitative trait locus in rice, is closely related to the Arabidopsis flowering time gene CONSTANS. The Plant cell. 2000;12(12):2473–84. . 11148291 10.1105/tpc.12.12.2473 PMC102231

[34] Martinez-Garcia JF, Virgos-Soler A, Prat S. Control of photoperiod-regulated tuberization in potato by the Arabidopsis flowering-time gene CONSTANS. Proc Natl Acad Sci U S A. 2002;99(23):15211–6. . 12393812 10.1073/pnas.222390599 PMC137569

[35] Carter TE Jr., R. N, C.H. S, Z. C. Genetic diversity in soybean Soybeans: Improvement, Production and Uses. eds Boerma HR SJ, editor: (Am Soc Agron, Madison, WI); 2004.

[36] Kim MY, Van K, Kang YJ, Kim KH, Lee SH. Tracing soybean domestication history: From nucleotide to genome. Breed Sci. 2012;61(5):445–52. 10.1270/jsbbs.61.445 23136484 PMC3406779

[37] Watanabe S, Harada K, Abe J. Genetic and molecular bases of photoperiod responses of flowering in soybean. Breed Sci. 2012;61(5):531–43. 10.1270/jsbbs.61.531 23136492 PMC3406791

[38] Liu H, Wang H, Gao P, Xu J, Xu T, Wang J, et al Analysis of clock gene homologs using unifoliolates as target organs in soybean (*Glycine max*). J Plant Physiol. 2009;166(3):278–89. 10.1016/j.jplph.2008.06.003 18707796

[39] Owen FV. Inheritance studies in soybeans. II. Glabrousness, color of pubescence, time of maturity, and linkage relations. Genetics. 1927;12:519–29. 17246537 10.1093/genetics/12.6.519 PMC1200963

[40] Bernard R. Two genes for time of flowering in soybeans. Crop Sci. 1971;11:242–4.

[41] Buzzell RI. Inheritance of a soybean flowering response to fluorescent-daylength conditions. Can J Cytol. 1971;13:703–7.

[42] Buzzell RI, Voldeng HD. Inheritance of insensitivity to long daylength. Soyb Genet Newsl. 1980;7:26–9.

[43] McBlain BA, Bernard RL. A new gene affecting the time of flowering and maturity in soybean. J Hered. 1987;78:160–2.

[44] Bonato ER, Vello NA. E6, a dominant gene conditioning early flowering and maturity in soybeans. Genet Mol Biol. 1999;22:229–32.

[45] Cober ER, Voldeng HD. A new soybean maturity and photoperiodsensitivity locus linked to E1 and T. Crop Sci. 2001;41:698–701.

[46] Cober ER, Molnar SJ, Charette M, Voldeng HD. A New Locus for Early Maturity in Soybean. Crop Science. 2010;50(2):524–7. 10.2135/cropsci2009.04.0174 .

[47] Kong FJ, Nan HY, Cao D, Li Y, Wu FF, Wang JL, et al A New Dominant Gene E9 Conditions Early Flowering and Maturity in Soybean. Crop Science. 2014;54(6):2529–35. 10.2135/cropsci2014.03.0228 .

[48] Ray JD, Hinson K, Manjono JE, Malo MF. Genetic control of longjuvenile trait in soybean. Crop Sci. 1995;35:1001–6.

[49] Xia Z, Watanabe S, Yamada T, Tsubokura Y, Nakashima H, Zhai H, et al Positional cloning and characterization reveal the molecular basis for soybean maturity locus E1 that regulates photoperiodic flowering. Proc Natl Acad Sci U S A. 2012;109(32):E2155–64. 10.1073/pnas.1117982109 22619331 PMC3420212

[50] Watanabe S, Xia Z, Hideshima R, Tsubokura Y, Sato S, Yamanaka N, et al A map-based cloning strategy employing a residual heterozygous line reveals that the GIGANTEA gene is involved in soybean maturity and flowering. Genetics. 2011;188(2):395–407. 10.1534/genetics.110.125062 21406680 PMC3122305

[51] Watanabe S, Hideshima R, Xia Z, Tsubokura Y, Sato S, Nakamoto Y, et al Map-based cloning of the gene associated with the soybean maturity locus E3. Genetics. 2009;182(4):1251–62. 10.1534/genetics.108.098772 19474204 PMC2728863

[52] Liu B, Kanazawa A, Matsumura H, Takahashi R, Harada K, Abe J. Genetic redundancy in soybean photoresponses associated with duplication of the phytochrome A gene. Genetics. 2008;180(2):995–1007. 10.1534/genetics.108.092742 18780733 PMC2567397

[53] Thomas B, Vince-Rue D. Photoperiodism in Plants Second Edition ed: pp. 11–13. Academic Press Limited, London; 1997a. 428 p.

[54] Li F, Zhang X, Hu R, Wu F, Ma J, Meng Y, et al Identification and Molecular Characterization of FKF1 and GI Homologous Genes in Soybean. PloS one. 2013;8(11):e79036 10.1371/journal.pone.0079036 24236086 PMC3827303

[55] Kardailsky I, Shukla VK, Ahn JH, Dagenais N, Christensen SK, Nguyen JT, et al Activation tagging of the floral inducer FT. Science. 1999;286(5446):1962–5. . 10583961 10.1126/science.286.5446.1962

[56] Matsoukas IG, Massiah AJ, Thomas B. Florigenic and antiflorigenic signaling in plants. Plant Cell Physiol. 2012;53(11):1827–42. 10.1093/pcp/pcs130 . 23008422

[57] Song YH, Ito S, Imaizumi T. Flowering time regulation: photoperiod- and temperature-sensing in leaves. Trends Plant Sci. 2013;18(10):575–83. 10.1016/j.tplants.2013.05.003 23790253 PMC3796012

[58] Hayama R, Coupland G. The molecular basis of diversity in the photoperiodic flowering responses of Arabidopsis and rice. Plant Physiol. 2004;135(2):677–84. . 15208414 10.1104/pp.104.042614 PMC514104

[59] Greenup A, Peacock WJ, Dennis ES, Trevaskis B. The molecular biology of seasonal flowering-responses in Arabidopsis and the cereals. Ann Bot. 2009;103(8):1165–72. 10.1093/aob/mcp063 19304997 PMC2685306

[60] Roulin A, Auer PL, Libault M, Schlueter J, Farmer A, May G, et al The fate of duplicated genes in a polyploid plant genome. Plant J. 2012 Epub 2012/09/15. 10.1111/tpj.12026 . 22974547

[61] Schmutz J, Cannon SB, Schlueter J, Ma J, Mitros T, Nelson W, et al Genome sequence of the palaeopolyploid soybean. Nature. 2010;463(7278):178–83. 10.1038/nature08670 20075913

[62] Schlueter JA, Lin JY, Schlueter SD, Vasylenko-Sanders IF, Deshpande S, Yi J, et al Gene duplication and paleopolyploidy in soybean and the implications for whole genome sequencing. BMC Genomics. 2007;8:330 . 17880721 10.1186/1471-2164-8-330 PMC2077340

[63] Yoo SJ, Hong SM, Jung HS, Ahn JH. The cotyledons produce sufficient FT protein to induce flowering: evidence from cotyledon micrografting in Arabidopsis. Plant Cell Physiol. 2013;54(1):119–28. 10.1093/pcp/pcs158 . 23204014

[64] Ogawa Y, King RW. The inhibition of flowering by non-induced cotyledons of *Parbitis nil* . Plant Cell Physiol. 1990;31:129–35.

[65] Paton DM. Photoperiodic induction of flowering in the late pea cultivar Greenfeast: the role of exposed cotyledons and leaves. Aust J Biol Sci. 1971;24:609–18.

[66] Jiang B, Nan H, Gao Y, Tang L, Yue Y, Lu S, et al Allelic combinations of soybean maturity Loci E1, E2, E3 and E4 result in diversity of maturity and adaptation to different latitudes. PloS one. 2014;9(8):e106042 10.1371/journal.pone.0106042 25162675 PMC4146597

[67] Zhai H, Lu S, Wang Y, Chen X, Ren H, Yang J, et al Allelic variations at four major maturity e genes and transcriptional abundance of the e1 gene are associated with flowering time and maturity of soybean cultivars. PloS one. 2014;9(5):e97636 10.1371/journal.pone.0097636 24830458 PMC4022622

[68] Tsubokura Y, Watanabe S, Xia Z, Kanamori H, Yamagata H, Kaga A, et al Natural variation in the genes responsible for maturity loci E1, E2, E3 and E4 in soybean. Ann Bot. 2014;113(3):429–41. 10.1093/aob/mct269 24284817 PMC3906962

[69] Langewisch T, Zhang H, Vincent R, Joshi T, Xu D, Bilyeu K. Major soybean maturity gene haplotypes revealed by SNPViz analysis of 72 sequenced soybean genomes. PloS one. 2014;9(4):e94150 10.1371/journal.pone.0094150 24727730 PMC3984090

[70] Thomas B, Vince-Rue D. Photoperiodism in Plants Second Edition ed: pp.355–365. Academic Press Limited, London; 1997b. 428 p.

[71] Hu R, Fan C, Li H, Zhang Q, Fu YF. Evaluation of putative reference genes for gene expression normalization in soybean by quantitative real-time RT-PCR. BMC Mol Biol. 2009;10:93 10.1186/1471-2199-10-93 19785741 PMC2761916

[72] Paz MM, Martinez JC, Kalvig AB, Fonger TM, Wang K. Improved cotyledonary node method using an alternative explant derived from mature seed for efficient Agrobacterium-mediated soybean transformation. Plant cell reports. 2006;25(3):206–13. 10.1007/s00299-005-0048-7 . 16249869

[73] Wang K. Agrobacterium-mediated transformation of soybean and recovery of transgenic soybean plants Iowa State University, Plant Transformation Facility. pp1-6. http://agron-www.agron.iastate.edu/ptf/service/Agrosoy.aspx2010 [updated 2010-3-22]. Available from: http://agron-www.agron.iastate.edu/ptf/service/Agrosoy.aspx.

[74] Olhoft PM, Donovan CM, Somers D. Soybean (Glycine max) transformation using mature cotyledonary node explants. Methods Mol Biol. 2006;343:385–96. 16988361 10.1385/1-59745-130-4:385

