# Supplementary material for: Extensive Analysis of GmFTL and GmCOL Expression in Northern Soybean Cultivars in Field Conditions
Source: PLoS One. 2015 Sep 15;10(9):e0136601. doi: 10.1371/journal.pone.0136601 (PMC4570765; doi:10.1371/journal.pone.0136601)
Supplement: S1 Dataset — (PDF) [file pone.0136601.s001.pdf]

S1 Dataset

All data including here are available upon request. However, the soybean seeds are not available because they are patented or belonging to GM materials which are limited to be distributed.
