# Supplementary material for: Extensive Analysis of GmFTL and GmCOL Expression in Northern Soybean Cultivars in Field Conditions
Source: PLoS One. 2015 Sep 15;10(9):e0136601. doi: 10.1371/journal.pone.0136601 (PMC4570765; doi:10.1371/journal.pone.0136601)
Supplement: S5 Fig — (PDF) [file pone.0136601.s006.pdf]

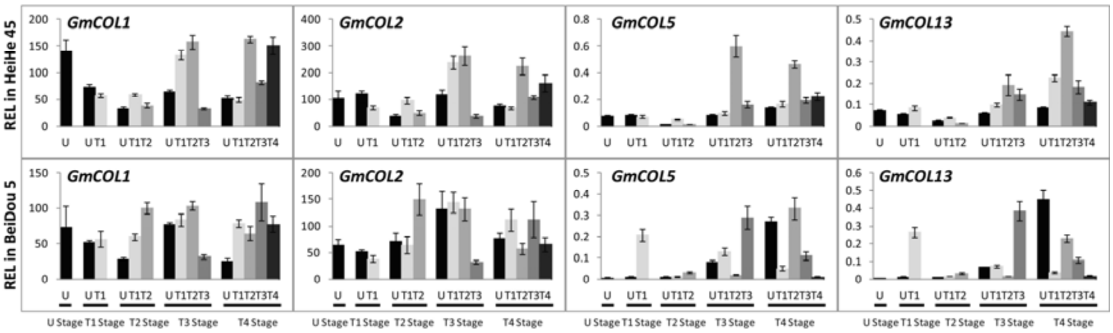

S5 Fig. Tissue-specific expression patterns of *GmCOL*s in soybean cultivars HeiHe 45 and BeiDou 5. Leaves: U, unifoliolate; T1, the 1st trifoliolate; T2, the 2nd trifoliolate; T3, the 3rd trifoliolate stage. U, U-stage,; T1, T1-stage; T2, T2-stage; T3, T3-stage; T4, T4-stage. The detailed information of stages and sampling were shown in the Materials and Methods section. Relative expression levels (REL) were analyzed by qRT-PCR and normalized to *UKN1*. Averages and standard errors are the result of three replicates.
