# Supplementary material for: Extensive Analysis of GmFTL and GmCOL Expression in Northern Soybean Cultivars in Field Conditions
Source: PLoS One. 2015 Sep 15;10(9):e0136601. doi: 10.1371/journal.pone.0136601 (PMC4570765; doi:10.1371/journal.pone.0136601)
Supplement: S9 Fig — (PDF) [file pone.0136601.s010.pdf]

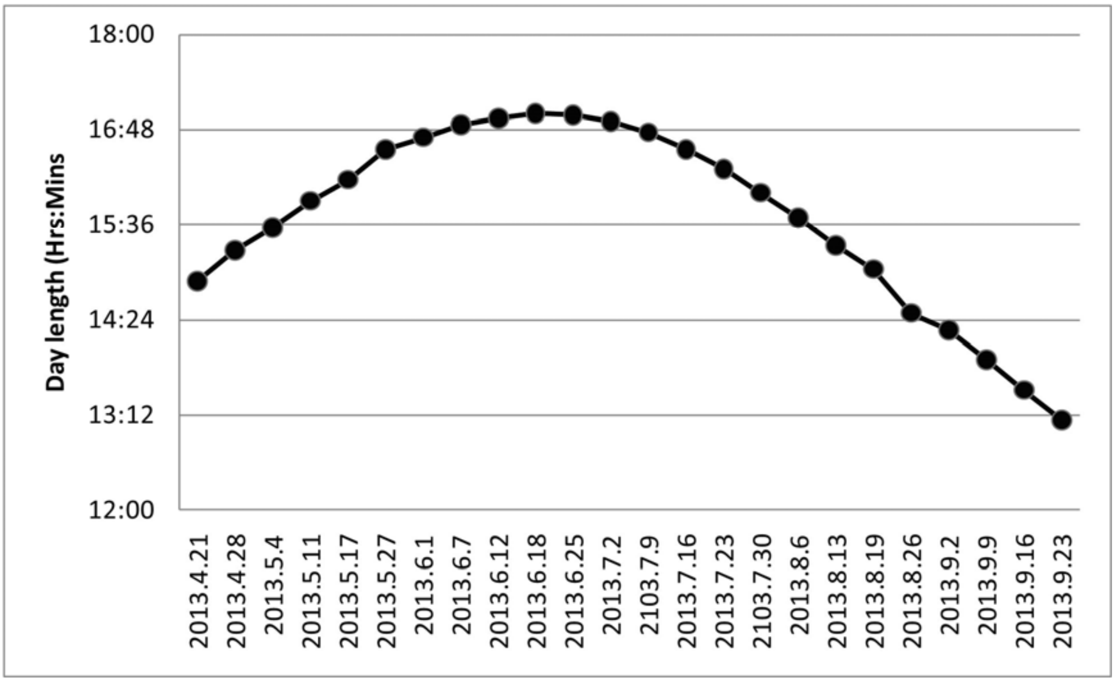

S9 Fig. The day-length change in Zhengzhou, Henan province, China, where all transgenic plants grew. The day length data are from the Era Shuttle Calendar (cf. Figure 1) (http://if.ustc.edu.cn/~ygwu/calendar.html).
