# Supplementary material for: Extensive Analysis of GmFTL and GmCOL Expression in Northern Soybean Cultivars in Field Conditions
Source: PLoS One. 2015 Sep 15;10(9):e0136601. doi: 10.1371/journal.pone.0136601 (PMC4570765; doi:10.1371/journal.pone.0136601)
Supplement: S10 Fig — (PDF) [file pone.0136601.s011.pdf]

A

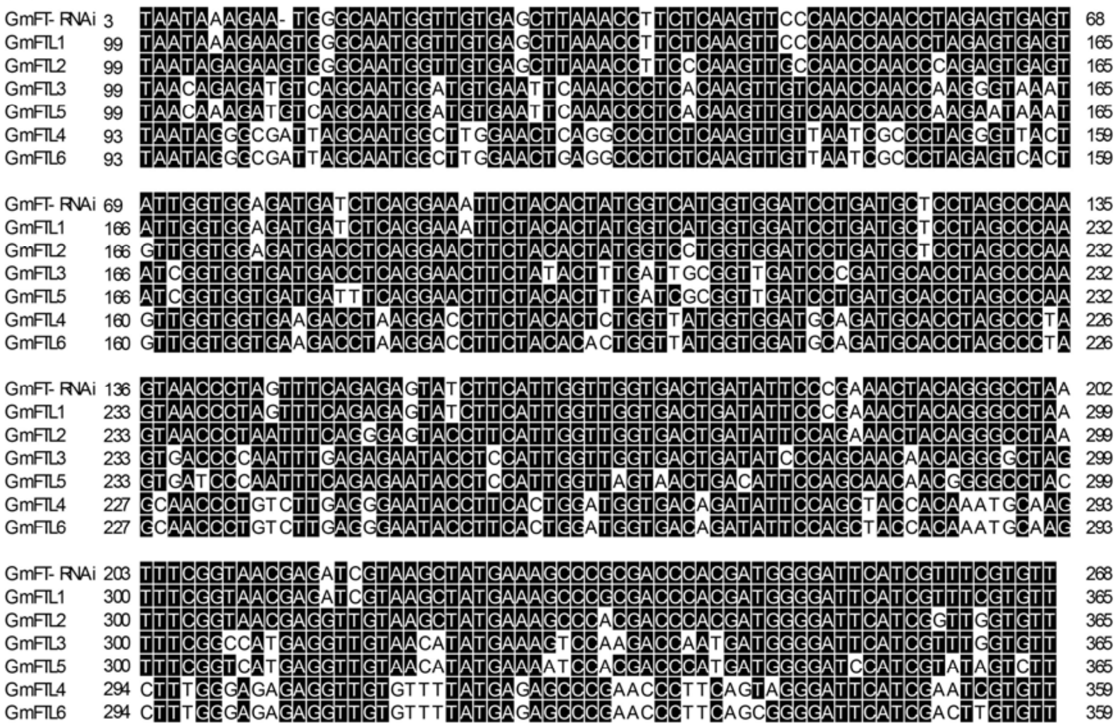

B

	GmFTL1	*GmFTL2*	*GmFTL3*	*GmFTL5*	*GmFTL4*	*GmFTL6*
Similarity	100.0	93.6	78.9	79.3	70.3	69.9
Avg. PCT. of knock-down	72.5	64.8	95.6	55.1	75.1	74.8

S10 Fig. The strategy and efficiency of *GmFTL* silencing. A, Alignment of the fragment of *GmFT*-RNAi and the coding sequences of *GmFTL*s. B, the efficiency of *GmFT*-RNAi. Similarity, the sequence similarity between *GmFT*-RNAi fragment of *GmFTL* genes; Avg. PCT, average percentage.
