# Supplementary material for: Extensive Analysis of GmFTL and GmCOL Expression in Northern Soybean Cultivars in Field Conditions
Source: PLoS One. 2015 Sep 15;10(9):e0136601. doi: 10.1371/journal.pone.0136601 (PMC4570765; doi:10.1371/journal.pone.0136601)
Supplement: S1 Table — (PDF) [file pone.0136601.s012.pdf]

S1 Table The names of *GmFTL*s in this study and literature

Locus name	Name in our lab	Name in other lab*
Glyma16g04840	*GmFTL1*	*GmFT3a*
Glyma19g28390	*GmFTL2*	*GmFT3b*
Glyma16g26660	*GmFTL3*	*GmFT2a*
Glyma16g04830	*GmFTL4*	*GmFT5a*
Glyma16g26690	*GmFTL5*	*GmFT2b*
Glyma19g28400	*GmFTL6*	*GmFT5b*
Glyma08g47820	*GmFTL7*	*GmFT6*
Glyma08g47810	*GmFTL8*	*GmFT4*
Glyma08g28470	*GmFTL9*	---
Glyma18g53690	*GmFTL10*	*GmFT1b*
Glyma18g53680	*GmFTL11*	*GmFT1a*

*Kong F., Liu B., Xia Z., Sato S., Kim B.M., Watanabe S., Yamada T., Tabata S., Kanazawa A., Harada K. & Abe J. (2010) Two coordinately regulated homologs of FLOWERING LOCUS T are involved in the control of photoperiodic flowering in soybean. *Plant Physiol*, **154**, 1220-1231.

"---" indicates not-identified in this study.
