# Supplementary material for: Extensive Analysis of GmFTL and GmCOL Expression in Northern Soybean Cultivars in Field Conditions
Source: PLoS One. 2015 Sep 15;10(9):e0136601. doi: 10.1371/journal.pone.0136601 (PMC4570765; doi:10.1371/journal.pone.0136601)
Supplement: S2 Table — (PDF) [file pone.0136601.s013.pdf]

S2 Table Sampling organs and date/time for gene expression analysis of circadian. The sampling time refers to China Standard Time.

Developmental Stage / Date	The type of leaves				
	Unifoliolate	1st trifoliolate	2nd trifoliolate	3rd trifoliolate	4th trifoliolate
Unifoliolate opening fully/ 05.27	4:00， 8:00<br>12:00， 16:00<br>20:00，24：00				
1st trifoliolate opening fully/ 06.01	4:00， 8:00<br>12:00， 16:00<br>20:00，24：00	4:00， 8:00<br>12:00， 16:00<br>20:00，24：00			
2nd trifoliolate opening fully/ 06.07	4:00， 8:00<br>12:00， 16:00<br>20:00，24：00	4:00， 8:00<br>12:00， 16:00<br>20:00，24：00	4:00， 8:00<br>12:00， 16:00<br>20:00，24：00		
3rd trifoliolate opening fully/ 06.12	4:00， 8:00<br>12:00， 16:00<br>20:00，24：00	4:00， 8:00<br>12:00， 16:00<br>20:00，24：00	4:00， 8:00<br>12:00， 16:00<br>20:00，24：00	4:00， 8:00<br>12:00， 16:00<br>20:00，24：00	
4th trifoliolate opening fully/ 06.18	4:00， 8:00<br>12:00， 16:00<br>20:00，24：00	4:00， 8:00<br>12:00， 16:00<br>20:00，24：00	4:00， 8:00<br>12:00， 16:00<br>20:00，24：00	4:00， 8:00<br>12:00， 16:00<br>20:00，24：00	4:00， 8:00<br>12:00， 16:00<br>20:00，24：00
