# Supplementary material for: Extensive Analysis of GmFTL and GmCOL Expression in Northern Soybean Cultivars in Field Conditions
Source: PLoS One. 2015 Sep 15;10(9):e0136601. doi: 10.1371/journal.pone.0136601 (PMC4570765; doi:10.1371/journal.pone.0136601)
Supplement: S3 Table — (PDF) [file pone.0136601.s014.pdf]

## S3 Table The list of primers used in this study

| Name of primer | Sequence (5' to 3') | Annotation and Functions |
|---|---|---|
| GmFTL4-AscⅠ-F | CACGTAGGCGCGCCATGGCACGGGAGAACCCTCT | construction of GmFTL4-CDS to pFGC5941 |
| GmFTL4-XbaⅠ-R | CACACATCTAGAATATCTCCTTCCACCGCAAC | construction of GmFTL4-CDS to pFGC5941 |
| FTL-RNAi -F | GGTAATAAAGAATGGGCAATGG | construction of FTL to pB7G |
| FTL-RNAi -R | CACAAACACGAAACGATGAATC | construction of FTL to pB7G |
| qGmFTL1-U111F | ATGAATATGTAACCGAAGCACTC | qRT-PCR for GmFTL1 |
| qGmFTL1-U275R | TGTTGACACTTGACAAACTCC | qRT-PCR for GmFTL1 |
| qGmFTL2-U5F | ATCTCTTCTGTTAATGTACCAAAAGTG | qRT-PCR for GmFTL2 |
| qGmFTL2-U110R | GTGACCCATGAGTGTTATTATATGTAG | qRT-PCR for GmFTL2 |
| qGmFTL3-505F | GGTTCTGGTGGAAGGAGGTTATAC | qRT-PCR for GmFTL3 |
| qGmFTL3-U96R | ACTACTAAAGAGTGTGGGAGATTGC | qRT-PCR for GmFTL3 |
| qGmFTL4-U138F | GGACAGAAGCAAAATTAAAGCAGATG | qRT-PCR for GmFTL4 |
| qGmFTL4-U21R | ACTATATACTATGATGTTTGTGTTTGGG | qRT-PCR for GmFTL4 |
| qGmFTL5-U128F | GCGACAAGCACAAGCGATTC | qRT-PCR for GmFTL5 |
| qGmFTL5-U29R | TGTTATGACACCAACTATGATGAGG | qRT-PCR for GmFTL5 |
| qGmFTL6-500F | GCGGTGGAAGGAGATATTAACAAG | qRT-PCR for GmFTL6 |
| qGmFTL6-U128R | ATCATCAGATCAAAGGGCATAGAC | qRT-PCR for GmFTL6 |
| UKN1-F | TGGTGCTGCCGCTATTTACTG | reference gene for qRT-PCR |
| UKN1-R | GGTGGAAGGAACTGCTAACAAT | reference gene for qRT-PCR |
| GFP-163-F | CCCGTGCCCTGGCCCACCCTCGTG | qRT-PCR for GFP |
| GFP-273-R | GATGGTGCGCTCCTGGACGTAGCCT | qRT-PCR for GFP |
| E1-314-qF | AATGAAGAGGAGATGAACAAT | qRT-PCR for E1 |
| E1-432- qR | AGCAAGAGTCTACTTAGGAT | qRT-PCR for E1 |
| E2-4135-qF | CTGGATAGAACAGACTTGTACAG | qRT-PCR for E2 |
| E2-4272-qR | GGAAATCCTGCCTGAACATAC | qRT-PCR for E2 |
| E3-676-qF | CCCTTCTCTCTGCTCATC | qRT-PCR for E3 |
| E3-778-qR | GTTCTAGATCTGCCAGAATT | qRT-PCR for E3 |
| E4-4110-qF | GATGATGCAACCAGCAAAAC | qRT-PCR for E4 |
| E4-4275-qR | GGCCACGGAATGGGAAGAAG | qRT-PCR for E4 |
| GI1-2844-qF | GGGCTGGAGACAGGTTGTTGATGC | qRT-PCR for GI1 |
| GI1-2978-qR | GTTAGTGGCAAACGTAATTTATG | qRT-PCR forGI1 |
| GI2--qF | GTAAACCTTCTCATTCTGCTAG | qRT-PCR forGI2 |
| GI2--qR | CATTGCTTGAAGTCGTGTTTGGG | qRT-PCR forGI2 |
